# Graphene Oxide-Gold Nanosheets Containing Chitosan Scaffold Improves Ventricular Contractility and Function After Implantation into Infarcted Heart

**DOI:** 10.1038/s41598-018-33144-0

**Published:** 2018-10-10

**Authors:** Sekaran Saravanan, Niketa Sareen, Ejlal Abu-El-Rub, Hend Ashour, Glen Lester Sequiera, Hania I. Ammar, Venkatraman Gopinath, Ashraf Ali Shamaa, Safinaz Salah Eldin Sayed, Meenal Moudgil, Jamuna Vadivelu, Sanjiv Dhingra

**Affiliations:** 1 Regenerative Medicine Program, Institute of Cardiovascular Sciences, St. Boniface Hospital Research Centre, Department of Physiology and Pathophysiology, University of Manitoba, Winnipeg, Canada; 2 Department of Physiology, Biochemistry and Histology, Faculty of Medicine, Cairo University, Cairo, Egypt; 3 Department of Medical Microbiology, Faculty of Medicine, University of Malaya, Kuala Lumpur, Malaysia; 4 Present Address: CeNTAB, Department of Bioengineering, SASTRA University, Thanjavur, Tamil Nadu, 613401 India

## Abstract

Abnormal conduction and improper electrical impulse propagation are common in heart after myocardial infarction (MI). The scar tissue is non-conductive therefore the electrical communication between adjacent cardiomyocytes is disrupted. In the current study, we synthesized and characterized a conductive biodegradable scaffold by incorporating graphene oxide gold nanosheets (GO-Au) into a clinically approved natural polymer chitosan (CS). Inclusion of GO-Au nanosheets in CS scaffold displayed two fold increase in electrical conductivity. The scaffold exhibited excellent porous architecture with desired swelling and controlled degradation properties. It also supported cell attachment and growth with no signs of discrete cytotoxicity. In a rat model of MI, *in vivo* as well as in isolated heart, the scaffold after 5 weeks of implantation showed a significant improvement in QRS interval which was associated with enhanced conduction velocity and contractility in the infarct zone by increasing connexin 43 levels. These results corroborate that implantation of novel conductive polymeric scaffold in the infarcted heart improved the cardiac contractility and restored ventricular function. Therefore, our approach may be useful in planning future strategies to construct clinically relevant conductive polymer patches for cardiac patients with conduction defects.

## Introduction

Myocardial infarction (MI) results in interruption of blood supply to the heart cells, thereby causing the cells to die and the heart ultimately loses its function^1^. The limited regenerative potential of heart muscle causes scar formation and abrogates normal electrical signal propagation and impairs conduction leading to delay or block in impulse propagation^2^. Electrical disturbances result in the loss of electrical coupling between healthy and scarred myocardial zone leading to functional decompensation. Apart from MI, conduction disorders such as atrioventricular (AV) block and ventricular arrhythmias also need clinical intervention for removing conduction blocks^3^. The conductive polymers are appealing biomaterials with the ability to promote electrical conduction in nonconductive areas and support cell growth *in vivo*^4^. Therefore, conductive polymers are excellent candidates which can mimic the native extracellular matrix (ECM) for cell growth and also preserve electrical coupling between cells of the myocardium in the infarcted heart. In recent years, application of conductive polymers to treat various disorders has gained much attention^5–9^.

The chitosan (CS) based scaffolds are widely studied for tissue engineering applications. Chitosan, a biocompatible, biodegradable and clinically accepted (FDA approved) polymer is being tested for cardiac tissue regeneration and repair as cell laden system or acellular matrice^10,11^. However, lack of electrical conductivity associated with chitosan based biomaterials had limited their application for treating conduction abnormalities of the heart and other organs. The addition of a conductive polymer or grafting of conductive nanoparticles in the base polymer can significantly improve electrical conductivity of the scaffold^12–14^. Recently, gold nanoparticles incorporated in the scaffolds were reported to promote electrical signal conduction in cardiomyocytes and neurons^12,15^. However, nanoparticles after incorporation into the scaffolds tend to aggregate into isolated microstructures, which might cause stress as well as damage to the surrounding tissue. Therefore, in this study, we engineered a novel conductive scaffold by using chitosan as a base biomaterial, and incorporating it with a conductive polymer, which was synthesized by anchoring gold nanoparticles (AuNPs) to graphene oxide (GO) sheets. We hypothesized that the addition of AuNPs into GO sheets, before incorporating it to the chitosan, will not only prevent aggregation of AuNPs in the chitosan microstructures, also it will facilitate homogenous and even distribution of AuNPs in the conductive polymer scaffold. Previous studies reported that GO promotes cell adhesion, proliferation, adsorption of ECM proteins and possess enhanced mechanical and electrical properties^16–18^. Therefore in addition to acting as a support material for AuNPs, graphene oxide sheets will also promote native cell growth and proliferation. Further, due to its high mechanical strength^19^ and electrical properties^20^, GO will provide enhanced mechanical strength to the chitosan scaffold and reduced electrical impedance. We characterized this novel conductive polymer scaffold, and found that it is not toxic to cell growth or metabolism, can improve cardiac contractility *ex-vivo* as well as *in vivo*, and lead to a significant improvement in cardiac function when implanted into rat model of myocardial infarction.

## Results and Discussion

### Synthesis and characterization of graphene oxide-gold nanosheet

**Figure 1:**
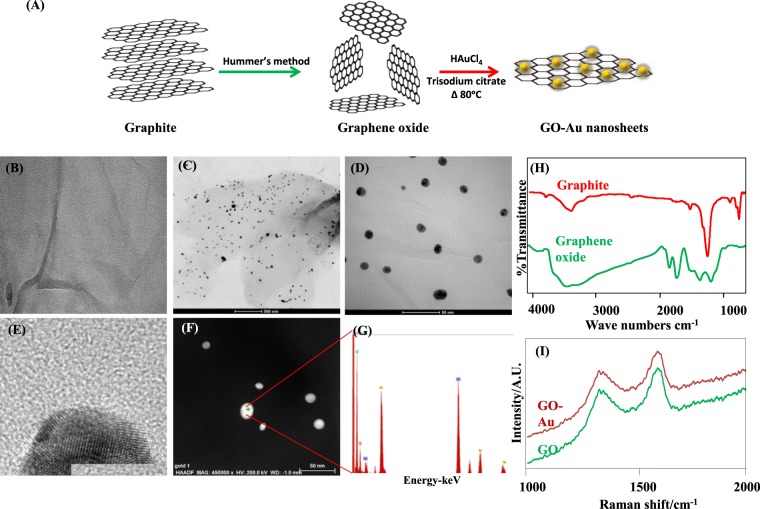
Synthesis and characterization of graphene oxide-gold nanosheets: (**A**) Diagrammatic representation of GO exfoliation by hummer’s method and GO-Au nanosheets synthesis. Yellow colored spheres represent gold nanoparticles which are anchored to GO. (**B**) Transmission electron micrograph of GO. (**C**,**D)** the TEM images of GO-Au nanosheets. (**E**) High resolution - TEM image of gold nanoparticle (~8 nm) anchored onto GO. (**F**) Micrograph depicts STEM analysis of gold nanoparticles. (**G**) Energy dispersive spectra (EDS) of gold nanoparticles. (**H**) Fourier transformed infrared (FT-IR) spectra recorded for graphene oxide and graphite particles. (**I**) Raman spectra of graphene oxide (GO) and gold nanoparticles anchored graphene oxide (GO-Au). (n = 4).

Graphite flakes were exfoliated by Hummer’s method and GO sheets were synthesized. The GO sheets were embedded with gold nanoparticles by thermal-reduction using trisodium citrate at 80 °C (Fig. 1A). The surface geometry of GO sheets (before and after gold nanoparticle addition) was assessed by transmission electron microscopy (TEM). Our data indicate the presence of ex-foliated layers of graphene oxide (Fig. 1B). The smooth and planar sheets clearly demonstrate the presence of high surface area with necessary two dimensional (2D) structure for gold nanoparticles packing. The gold nanoparticles (AuNPs) loaded onto GO sheets (in TEM images) were anchored into the graphene oxide much evenly (Fig. 1C,D). The scanning transmission electron microscopy (STEM) analysis confirmed that the size of the AuNPs embedded in GO sheets was around 8 nm (Fig. 1E,F). Energy dispersive spectra (EDS) of the biomaterial were recorded for the confirmation of elemental composition of the GO-AuNPs composite. The elemental data demonstrated that the deposited nanoparticles on GO sheets are AuNPs as it was clearly indicated by the peaks denoting the presence of carbon (C), oxygen (O) and gold (Au) (Fig. 1G).

Exfoliation of graphene oxide sheets from graphite generally results in generation of numerous functional moieties such as carboxylic and carbonyl groups present in the structure of graphene oxide. The fourier transformed infrared (FT-IR) spectra of graphite and graphene oxide as presented in Fig. 1H clearly demonstrated oxidation in the presence of several bands attributed to oxygen functionalization in graphene oxide. The absorption peak at 1725 cm^−1^ and 1630 cm^−1^ depict C=O stretching of carbonyl and carboxylic groups. Absorption peaks at 1230 cm^−1^ and 1040 cm^−1^ correspond to C-O stretching vibration. The C=O groups are involved in the conjugation of biomolecules and various nanoparticles including gold through covalent or electrostatic interactions suited for various applications.

The attachment of AuNPs onto the highly flat GO sheets surface may have been mediated via electrostatic attraction of Au ions to oxygen functionalities on the GO surface. The high surface area of GO sheets make uniform loading of AuNPs. The structural changes in GO after gold nanoparticles loading were assessed by Raman spectroscopic measurements (Fig. 1I). Both naïve GO and GO-Au exhibited similar Raman spectra with typical D band (sp^3^-hybridized carbon) at 1352 cm^−1^ and a G band (tangential stretch) at 1599 cm^−1^. The ratio of D/G bands determines the structural deformities in the GO planes. In the current study, we found that anchoring AuNPs in GO planes had minor influence on the I_D_/I_G_ ratio. The I_D_/I_G_ ratio of pure GO was calculated to be 0.9 and GO-Au composite exhibited I_D_/I_G_ ratio of 0.86. Therefore, no structural distortion was detected in GO after gold nanoparticles addition^21,22^.

### Synthesis and interaction of GO-Au with chitosan

**Figure 2:**
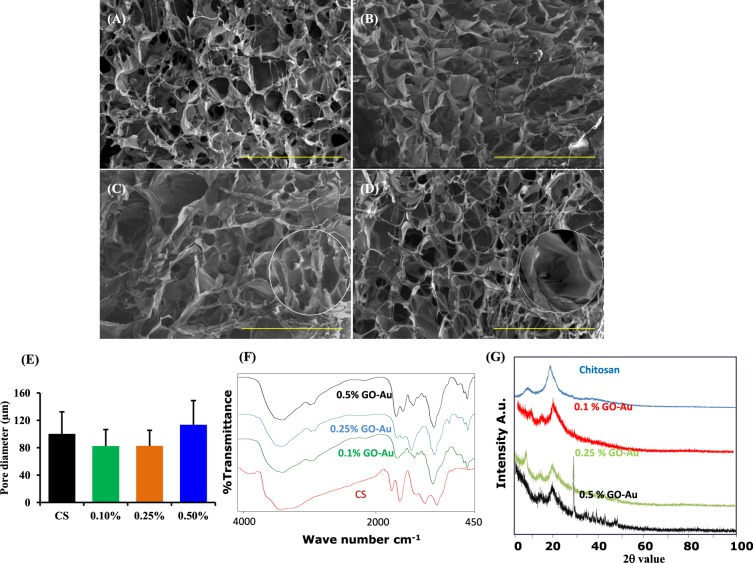
Physico-chemical characterization of the biocomposite scaffolds: (**A**–**D**) Scanning electron microscopy (SEM) images of the biocomposite scaffolds. SEM micrographs were captured for (**A**) Naïve chitosan (CS) scaffold, (**B**) CS/0.1% GO-Au, (**C**) CS/0.25% GO-Au and (**D**) CS/0.5% GO-Au scaffold. (**E**) Pore diameter of chitosan scaffolds at different concentrations of GO-Au (0.1%, 0.25%, 0.5%) was measured by image J software. (Scale bar = 500 µm). (**F**) FT-IR spectrum analysis of chitosan and scaffolds at different concentrations of GO-Au (0.1%, 0.25% and 0.5%). (n = 4–6). Data are expressed as mean ± SD.

Chitosan has been used extensively as a biomaterial for tissue engineering over the past two decades because of its biodegradable nature and porous structure^23,24^. The porosity of base biomaterial to be employed for tissue engineering applications plays a very important role^25^. The porous structure of the scaffold facilitates water or fluid adsorption from the surrounding tissue which is important for conductive nature of the material^14^. To optimize the concentration of GO-Au nanosheets to be added to chitosan to retain ideal porous nature of the chitosan scaffold, we systematically tested different concentrations of GO-Au (0.1%, 0.25, 0.5%, 0.75% and 1%) to a fixed concentration of chitosan (2%). The scaffolds were fabricated by freeze drying method in order to achieve a porous architecture. We assessed the porosity, pore-interconnectivity and pore dimensions of the scaffold by scanning electron microscopic (SEM) analysis. At 0.75% and 1% of GO-Au concentration there was a complete agglutination of pores and GO-Au polymer was not dispersed homogenously (data not shown). However, as demonstrated in our SEM pictures (Fig. 2A–D), at lower concentrations (0.1%, 0.25% and 0.5%), the dispersion of GO-Au was homogenous and we observed a well-developed network at these concentrations. The addition of GO-Au at these concentrations (0.1%, 0.25%, and 0.5%) had no significant effect on porous nature and pore size of the chitosan scaffold (Fig. 2E). The pore dimensions in the chitosan scaffold (with or without GO-Au) were in the range of 60–140 µm which is the ideal pore size for any biomaterial to be used for tissue engineering applications^26,27^.

To study the interaction between chitosan and GO-Au, we performed FTIR spectra for GO-Au and Chitosan-GO-Au composite scaffolds having different concentrations (0.1%, 0.25% and 0.5%) of GO-Au (Fig. 2F). In GO spectrum, the peak at 1035 cm^−1^ corresponds to the C-O-C bonds stretching vibration of epoxy groups and C-OH bonds are observed at 1230 cm^−1^ absorption bands. The peak observed at 1740 cm^−1^ is attributed to the C=O in carbonyl and carboxyl moieties of GO^28^. Chitosan exhibits major peaks at 1035 cm^−1^ and 1560 cm^−1^ corresponding to the absorbance of glycosidic bonds C=O stretch vibration (amide I and III) and N-H bonding of amino groups in its structure. The FT-IR spectra of the composite scaffolds with varied amounts of GO-Au nanosheets demonstrated that there is a shift in the amide groups from 1560 to 1580 cm^−1^ which clearly revealed the interactions between amide groups in CS and carboxylic groups in GO-Au with newly formed amide bonds between GO-Au nanosheets and chitosan^29,30^. Therefore, our data depict that GO-Au sheets were attached to chitosan matrix via amide linkage of GO carboxylic acid and amine group of chitosan forming amide bond between GO and chitosan. The presence of oxygen-containing groups on GO is observed in the spectra. Notably, most of the absorbance peaks in chitosan group and CS-GO-Au are overlapped.

### Improved physico-mechanical properties of the scaffold upon GO-Au inclusion into chitosan matrix

**Figure 3:**
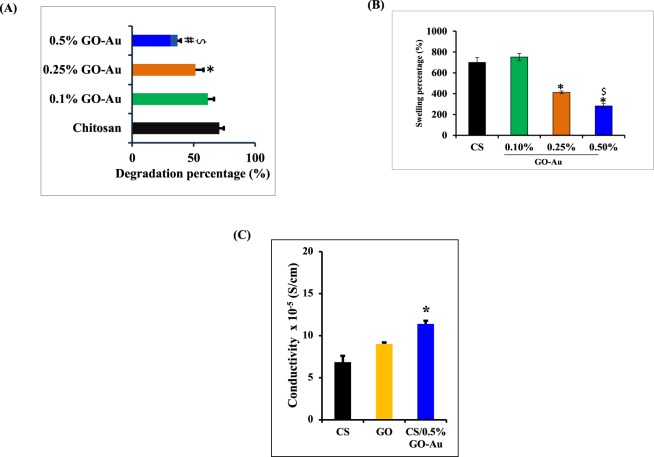
Physico-mechanical characterization of the composite scaffolds: (**A**) Degradation rate of the scaffolds in lysozyme containing medium was measured for 5weeks at 37 °C. Replenishment of fresh solution was carried out after every three days. Addition of GO-Au decreased the degradation rate of chitosan. At 0.1% GO-Au concentration, there was a decreasing trend, however the addition of GO-Au at 0.25% and 0.5% significantly slowed down the degradation rate compared to naïve chitosan. (**B)** Swelling percentage of the scaffolds was measured by immersing the scaffold in PBS for 24 h, our data indicated that addition of GO-Au at 0.25% and 0.5% concentrations significantly decreased the water retention ability of the scaffold. (**C**) The electrical conductivity of the scaffold was measured by Four Probe method. Addition of GO (at a concentration of 0.5%) did not cause a significant increase in the conductivity of scaffold. However, incorporation of GO-Au nanosheets at 0.5% concentration significantly improved the electrical conductivity of the scaffold compared to naïve chitosan. *P < 0.05 compared to control scaffold (CS) group. ^$^P < 0.05 -indicates significant decrease in CS/0.5% GO-Au group compared to other concentrations of GO-Au (0.1% and 0.25%) in the scaffold. (n = 4–6). Data are expressed as mean ± SD.

One of the major advantages of biomaterials based scaffolds is that they degrade *in vivo*, which is mediated by native hydrolytic enzymes in the body^31^. However, on the other hand, the retention of implanted material inside the body for desired amount of time is also essential to mediate its biological effects. Therefore, post-implantation degradation of the material should take place in a controlled manner. Biodegradation of porous scaffolds is dependent on several factors such as pore size, surface area, hydrophilicity and addition of co-polymers^32^. Chitosan is a polymer, bearing amino groups and breakable glycosidic bonds. It is mainly targeted by lysozyme, which hydrolyses glycosidic linkages in the polymer. Hence, in the current study, to evaluate the effect of GO-Au addition on the degradation rate of chitosan-GO-Au scaffold, the chitosan scaffolds containing different percentage of GO-Au were incubated with lysozyme containing medium for a period of 5 weeks and the degradation rate was calculated by measuring the final weight of the material. The addition of GO-Au slowed down the degradation of the biomaterial. At 0.1% of GO-Au, we observed a decreasing trend (even though it was not statistically significant) in degradation of the scaffold, however at 0.25% and 0.5% concentrations of GO-Au, the degradation rate was significantly reduced in a dose dependent manner compared to naïve chitosan scaffold (Fig. 3A). The decreased degradation rate of scaffolds with an increase in GO-Au content is probably due to the electrostatic interactions at hydrogen bonds among GO-Au nanosheets and CS matrix which strengthens the CS scaffold and enhances its stability^33^. Furthermore, GO-Au sheets hinder the penetration of hydrolytic enzymes inside the polymer matrix that slows down the degradation^34^.

Chitosan is a hydrophilic polymer with the ability to hydrate itself via protonation of amino groups^35^. Therefore, after *in vivo* implantation, the absorption of water or tissue fluids from the surrounding microenvironment results in swelling of the chitosan scaffold. However, the swollen scaffold would lead to wall stress to the surrounding tissue. Furthermore, excessive swelling of the scaffold limits it’s electrical conductivity, as the swelling of the network results in leaching out of conductive polymers from the scaffold^36^. Also, the swelling of the biomaterial exposes it to hydrolytic enzymes mediated degradation. Therefore, to achieve desired persistence of the biomaterial inside the body, a balance between swelling ability and degradation rate is very important. We evaluated the swelling properties of the polymer scaffold by immersing it in phosphate buffer saline (PBS). At 0.1% concentration of GO-Au, there was no significant difference observed in the swelling rate of the chitosan, however the addition of GO-Au at 0.25% and 0.5% concentrations significantly decreased the swelling rate of the scaffold (Fig. 3B). This behavior is attributed to the lesser hydrophilicity of GO compared to chitosan The pyranose rings in GO offer less space for such molecular transfer and water penetration^34,37^. Hence, increase in GO-Au content in our studies lead to decreased swelling ability of the scaffold.

Therefore, addition of GO-Au at lower concentrations (0.25%, 0.5%) led to a significant decrease in the degradation rate of the scaffold in a dose dependent manner. Also at these concentrations, with an increase in GO-Au content, the swelling percentage of the scaffold decreased. However, the addition of GO-Au at higher concentrations (0.75% and 1%) resulted in precipitate formation and disruption of 3D porous architecture of the chitosan scaffold. Therefore, to achieve controlled swelling ability and degradation rate of the polymeric scaffold, for rest of the *in vitro* experiments and *in vivo* studies, we used 0.5% of GO-Au in a fixed concentration of chitosan (2%).

The electrical conductivity of naïve chitosan, chitosan incorporated with 0.5% GO and chitosan incorporated with 0.5% GO-Au was measured by four-probe method with 2 contact pairs. Our data revealed that there was an increasing trend in the conductivity of scaffold with the inclusion of GO at a concentration of 0.5%, but this was not statistically significant. However, addition of 0.5% GO-Au led to a significant increase in the conductivity of chitosan scaffold (Fig. 3C). The conductive polymers are a special class of biomaterials which facilitate the direct delivery of electrical, electrochemical and electromechanical stimulus to cells. The conductive polymers are either used as independent base polymers or doped inside other non-conductive polymers to impart electrical conductivity. The conductivity of these materials arise from their unique structure of conjugated backbone alternated by single and double bonds in between which allows the movement of electrons with in the polymeric chains^38^. The gold nanoparticles, graphene nanosheets, carbon nanotubes are used as dopant to provide the electrical properties^12,14,15,39^. The gold nanoparticles (AuNPs) have been profoundly investigated for their tissue engineering applications^15,39,40^. They have been used to impart electrical properties to the non-conductive biomaterial based scaffolds and hydrogels^12,41^.

### Biocompatibility of the scaffold

Biomaterials intended for *in vivo* applications shouldn’t exert any cytotoxicity and should possess the ability to support cell growth^25^. Therefore, before proceeding to the *in vivo* experiments, we tested bio-compatibility of the conductive polymer scaffold. To investigate a wide range applicability of this novel biomaterial, we employed rat smooth muscle cells (SMCs), mouse fibroblasts and human induced pluripotent stem (iPS) cells derived cardiomyocytes for bio-compatibility assays.

**Figure 4:**
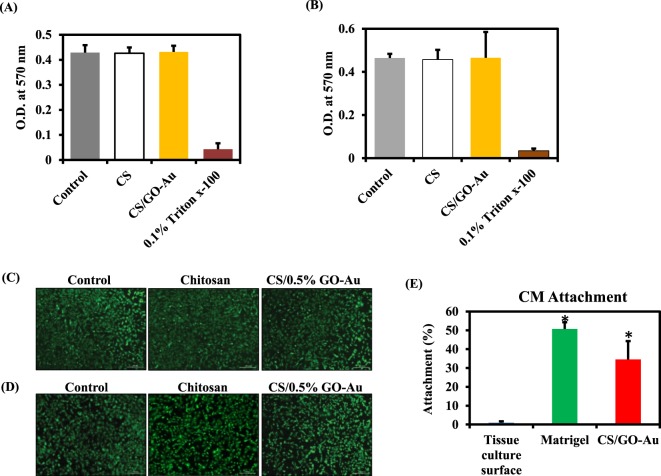
Biocompatibility of the scaffold was tested with rat smooth muscle cells, mouse fibroblasts and human iPSC derived cardiomyocytes: (**A**,**B**) Rat SMCs (**A**) and mouse fibroblasts (**B**) were grown on tissue culture plate (control), chitosan alone (CS) and chitosan-GO-Au for a period of 48 hr and assessed for metabolic activity by MTT assay. Cells treated with 0.1% triton X-100 were used as positive control. The scaffold supported the growth and metabolism of the cells without causing any cytotoxicity. (**C**,**D**) Rat SMC (**C**) and mouse fibroblasts (**D**) were grown on the tissue culture dish, naïve chitosan and CS-GO-Au scaffold film for 48 hr and stained with fluorescein diacetate (FDA). Cells exhibited well spread morphology on all the plates with cytoplasmic extensions. (Scale bar 300 µm). (**E**) Human iPSC derived cardiomyocytes (CMs) were cultured on normal tissue culture plates, matrigel coated plates, and CS-GO-Au scaffold. The cells were allowed to attach and cultured for 48 hr. The attachment percentage was calculated by manual counting under phase contrast microscope. The scaffold was compatible with human cardiomyocytes, as there was significant increase in the number of iPSC derived cardiomyocytes attached on CS-GO-Au scaffold and matrigel (positive control) compared to tissue culture plates after 48 hr of culture. *P < 0.05 compared to standard tissue culture plate. (n = 6). Data are expressed as mean ± SD.

Rat SMCs and mouse fibroblasts were grown on normal culture plates, chitosan, and Chitosan-GO-Au. The cells treated with 0.1% triton X-100 for 48 hr served a positive control. After 48 hr of incubation MTT assay was performed to assess growth of the cells. The smooth muscle cells and fibroblasts grown on chitosan and chitosan-GO-Au exhibited no significant reduction in growth and metabolic activity compared to the cells grown on normal culture plates (Fig. 4A,B). These results demonstrate that scaffold was compatible with rat SMCs and mouse fibroblasts. We also performed fluorescein diacetate (FDA) staining to assess viability of cells in the presence of scaffold. The data from FDA staining experiments complimented the results obtained from MTT assay, the cells after 48 hr of culture on normal culture plates, chitosan and chitosan-GO-Au were healthy and exhibited spindle shaped morphology, suggesting the compatibility of the biomaterial to both SMCs and fibroblasts (Fig. 4C,D).

To assess translational potential of conductive polymer scaffold, we investigated its biocompatibility with human induced pluripotent stem cells (iPSC) derived cardiomyocytes. iPSC derived cardiomyocytes (iPSC-CMs) were plated at a density of 3000 cells/well in a 96 well plate on different surfaces – normal tissue culture plates, matrigel coated plates, and chitosan-GO-Au scaffold. The cells were allowed to attach and culture for 48 hr. We found that the scaffold was compatible with human cardiomyocytes, as there was significant increase in the number of iPSC derived cardiomyocytes attached to chitosan-GO-Au scaffold and matrigel (positive control) compared to tissue culture plates after 48 hr of culture (Fig. 4E).

### Conductive polymer scaffold implantation improved the cardiac contractility in a rat model of MI

Previously published data demonstrate that biodegradable acellular scaffolds or stem cell laden scaffolds after implantation into the scar area in the infarcted heart lead to improvement in cardiac function by preventing ventricular dilation and enhancing myocardial regeneration^42,43^. However, the contraction and relaxation of the heart muscle depends on the conduction of electrical impulses through cardiac tissue. The conduction velocity is reduced and repolarization is delayed in the infarcted heart following a myocardial infarction^44^. Some of the recent studies evaluated the beneficial effects of injectable hydrogels on electrical conduction in the infarcted myocardium^5,12,45^. However, no previous studies have assessed the effects of an electrically conductive acellular scaffold to promote electrical propagation across an infarct area in the heart. We hypothesized that a conductive polymer scaffold after implantation into an infarcted heart will promote improvement in ventricular contraction and preserve heart function following an MI.

**Figure 5:**
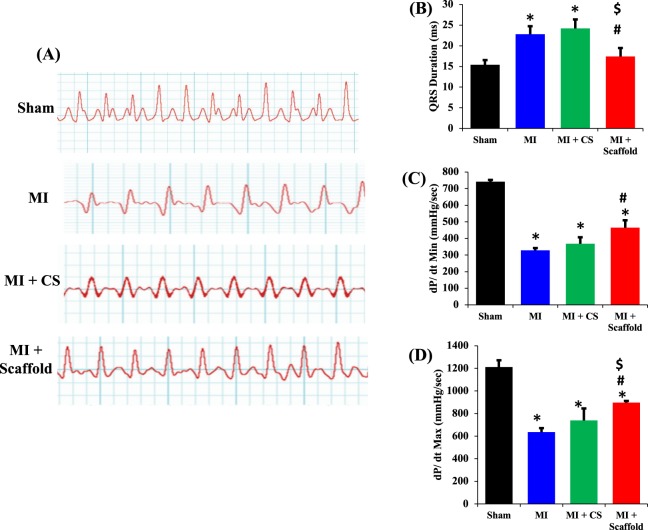
Cardiac contractility measurements *in vivo* and *ex vivo*: Cardiac contractility was measured by ECG and Langendorff after 5 weeks of scaffold implantation. Scaffold was implanted following a myocardial infarction. Electrocardiograms were recorded in conscious rats by non-invasive methods to avoid effect of anesthesia. (**A**) ECG measurements were performed after 5 weeks of MI. Coronary artery ligation significantly increased QRS interval. The implantation of only chitosan (MI + CS group) was not able to improve QRS duration, however conductive polymer scaffold (MI + scaffold group) implantation significantly improved the QRS intervals in MI induced hearts. (**B**) Histograms showing QRS interval duration. (**C**,**D**) At the end of *in vivo* experiments, the animals were anaesthetized and the hearts were removed and perfused on a Langendorff apparatus. The minimum and maximum rate of pressure rise dp/dt min and dp/dt max (two sensitive indices for contractility) were calculated. Both dp/dt min and dp/dt max decreased in MI groups, The implantation of only chitosan was not able to increase dp/dt min and dp/dt max, however conductive polymer scaffold implantation led to a significant increase in dP/dt min (**C**) and dP/dt max (**D**). *P < 0.05 compared to sham group, ^#^P < 0.05 compared to MI group, ^$^P < 0.05 compared to MI + CS group. (n = 5) Data are expressed as mean ± SD.

To determine whether implantation of the chitosan-GO-Au scaffold is beneficial in improving conduction velocity and cardiac contraction in the infarcted heart, rats were subjected to coronary artery ligation and the scaffold was implanted immediately after MI. The electrocardiogram (ECG) measurements were performed at 5 weeks (after scaffold implantation) (Fig. 5A). The ECG measurement is a very good indicator of conduction velocity and cardiac contraction. The time interval between QRS complex of ECG determines the cardiac rate or contraction. The shape of the QRS complex also changes when there is abnormal conduction of electrical impulses within the ventricles. If the QRS complex is prolonged, the conduction is impaired within the ventricles. In our studies, left coronary artery ligation (MI group) lead to prolongation of QRS intervals after 5 weeks of ligation compared to sham group. After 5 weeks of chitosan-GO-Au scaffold implantation, we observed a marked improvement in the mean QRS intervals, as there was a significant reduction in QRS complex. (Fig. 5B). Narrower QRS interval indicates more efficient conduction in the myocardium^46,47^. Interestingly, the implantation of chitosan scaffold (without GO-Au) did not improve mean QRS interval. Therefore, presence of GO-Au in the scaffold increased conductivity of the scaffold which might have been responsible for improvement in conduction velocity and contractility in the infarcted myocardium.

To further confirm that improvement in cardiac contractility is due to the implantation of conductive polymer scaffold and it is not mediated by any systemic parameters, the hearts from control group (sham), MI group, chitosan implanted group and chitosan-GO-Au scaffold implanted group were excised at the end of the study and perfused *ex-vivo* (by Langendorff apparatus). To measure cardiac contractility, the rate of change of ventricular pressure with respect to time (dP/dt) were calculated. It is already reported that maximum (dp/dt max) and minimum (dp/dt min) rate of pressure rise are direct indicators of ventricular contractility. In MI group there was a significant decrease in both dP/dt max and min after coronary artery ligation. Implantation of chitosan (without GO-Au) did not cause any change in dP/dt max and min compered to MI group. However, chitosan-GO-Au scaffold implantation significantly increased *ex-vivo* ventricular contractility, as there was a significant increase in dp/dt max and min in chitosan-GO-Au group compared to MI induced animals (Fig. 5C,D) These results confirm that conductive polymer scaffold implantation improved the efficiency of cardiac conduction following a cardiac injury. To the best of our knowledge, this is the first study reporting the application of a graphene oxide–gold nanoparticles incorporated chitosan scaffold in improving conduction velocity and contractility in the infarct area following a myocardial infarction.

### Effect of scaffold implantation on cardiac function in the infarcted heart

**Figure 6:**
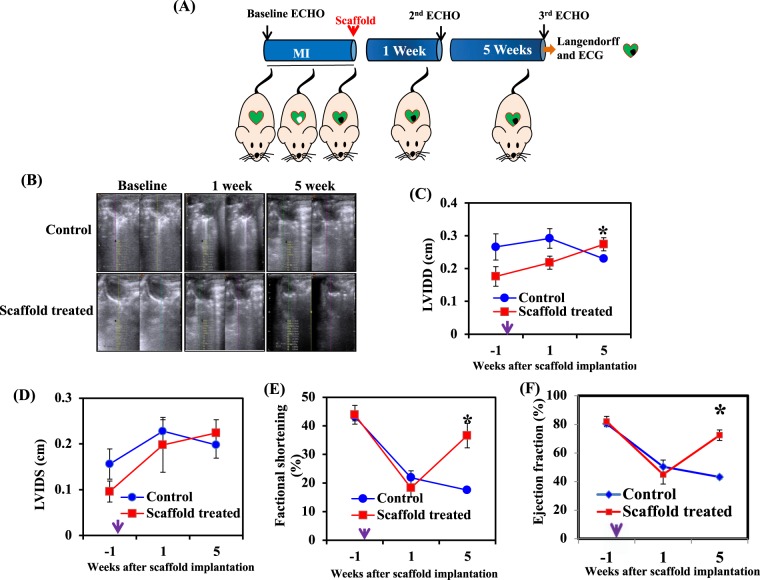
Implantation of conductive polymeric scaffold significantly improved the cardiac function in infarcted heart: Cardiac function were assessed by echocardiography at baseline, 1 week and 5 weeks (after scaffold implantation). (**A**) Graphical representation of MI model and time points for scaffold implantation. (**B**) Representative M-mode images of different groups. (**C**) Left ventricular internal dimension in diastole (LVIDD). (**D**) Left ventricular internal dimension in systole (LVIDS). (**E**) Percent fractional shortening (%FS). (**F**) Percent ejection fraction (%EF). Arrow in the x-axis indicates the time of scaffold implantation. *P < 0.05 compared to control group (MI group without scaffold). (n = 5). Data is expressed as mean ± SD.

To investigate the ability of chitosan-GO-Au scaffold in improving heart function following a myocardial infarction, rats were subjected to coronary artery ligation and the scaffold was implanted immediately after MI. Serial echocardiographic (ECHO) measurements were performed at base-line, 1 week and 5 weeks (after scaffold implantation) (Fig. 6A). Left coronary artery ligation resulted in significant LV dilatation and progressive deterioration of ventricular function, as assessed by ECHO (Fig. 6B–F). Interestingly, 1week after scaffold implantation left ventricular diameters %FS and % EF were not significantly different in control group and scaffold implanted group. However, after 5 weeks of scaffold implantation, we observed a significant improvement in cardiac function as there was a marked increase in %FS, %EF and left ventricular diameter dimensions (Fig. 6B–F). The increase in fractional shortening and ejection fraction in response to conductive polymer scaffold implantation clearly depicts the improvement in cardiac contractility and ability of the heart to pump blood in a more efficient manner. The observed improvement in contractility and QRS duration could be due to enhanced electrical coupling of scar area and adjacent tissue mediated by implanted biomaterial. The use of biodegradable scaffolds to preserve function in damaged ventricles and prevent heart failure is being explored in the clinic^10^. The application of conductive polymer scaffolds such as chitosan-GO-Au may represent an improvement over nonconductive scaffolds currently being tested, especially in patients with a wide QRS complex and delayed regional contraction due to MI.

### Implantation of conductive polymer scaffold did not induce any immune response in the host myocardium

**Figure 7:**
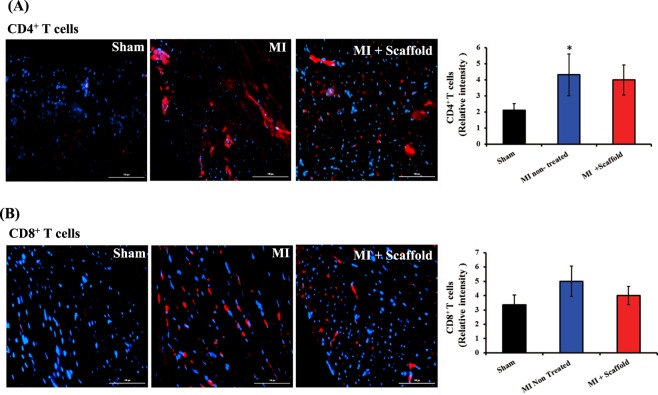
Implantation of conductive polymer patch did not induce host immune response *in vivo*: (**A**,**B**) Host immune response against implanted scaffold was assessed by evaluating CD4^+^ and CD8^+^ T cell infiltration in the myocardium by immunohistochemistry. The scaffold implantation did not cause any significant change in the number of infiltrating CD4^+^ and CD8^+^ T cells in the myocardium compared to MI group. *P < 0.05 compared to sham group. (n = 5). Data is expressed as mean ± SD.

We analyzed the *in vivo* host immune response against scaffold after 5 weeks of implantation. There was significant increase in the number of CD4^+^ and CD8^+^ T cells in the MI induced animals compared to sham group (Fig. 7A,B). The recruitment of T cells in the injured heart following a myocardial infarction is a dynamic process that helps in healing of myocardium and ventricular remodeling. Therefore we detected significant number of T cells in MI induced hearts. However, another reason for immune cell infiltration in the heart could be immune reaction to implanted material. Therefore, we compared the number of T cells in MI induced animals and MI + scaffold group. Our results demonstrate that scaffold implantation did not cause any significant change in the number of infiltrating CD4^+^ and CD8^+^ T cells in the myocardium compared to MI group (Fig. 7A,B). Therefore, the scaffold is biocompatible *in vivo* and it did not induce any immune responses in the heart.

### Conductive polymer scaffold implantation increased connexin 43 expression

**Figure 8:**
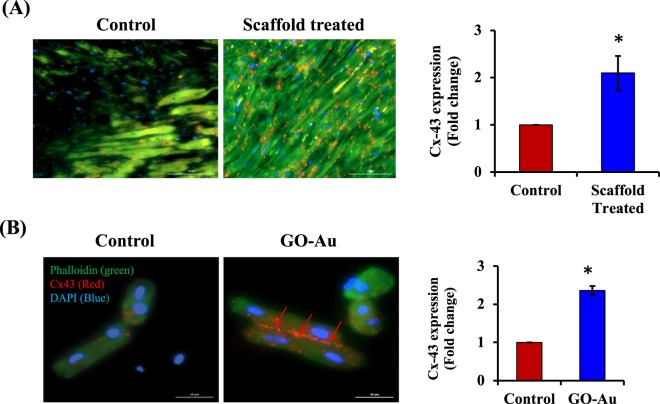
Connexin 43 expression in myocardial tissue and isolated cardiomyocytes: (**A**) Photomicrographs of rat myocardial sections (immunohistochemistry) in control group (MI) and scaffold (CS-GO-Au) treated group. Connexin 43 expression increased in scaffold treated animals compared to control group. (Scale bar = 100 µm). (F-actin-green; Connexin 43-red; DAPI-blue) (**B**) Connexin 43 expression (red arrows) in isolated cardiomyocytes (by immunocytochemistry) increased in GO-Au treated cells compared to control group. (Scale bars = 30 µm). (Phalloidin-green; Connexin 43-red; DAPI-blue). *P < 0.05 compared to respective control group. (n = 5–6). Data is expressed as mean ± SD).

In the next set of experiments, we wanted to explore the mechanisms of scaffold mediated improvement in electrical conduction and ventricular function^48^. Previously, it has been reported that reduced intercellular coupling and discontinuities in the cardiac tissue architecture are known to impede conduction through scar tissue in the infarcted heart^48,49^. In the myocardium, cardiomyocytes are arranged as interconnected functional syncytium to conduct electrical signals in the cardiac tissue. The major factors controlling intercellular coupling and electrical conduction are gap junction proteins. Connexin43 (Cx43) is the predominant ventricular gap junction protein, which is responsible for electrical conduction in the myocardium^50^. The pharmacological inhibition of Cx43 is reported to be associated with down regulation of electrical conduction in the myocardium^50^. Furthermore, genetic knockout of Cx43 in mice is associated with QRS prolongation, slow conduction and increased susceptibility to ventricular arrhythmias^51^. Our immunohistochemistry data (Fig. 8A; Supplementary Fig. 1) revealed that after conductive polymer scaffold implantation, there was a significant increase in Cx43 expression in the infarcted myocardium compared to control group (MI group). Connexin 43 is the most predominant protein present in ventricular cardiomyocyte gap junctions, and it helps in the propagation of intracellular electrical conduction. Therefore, we also assessed Cx43 expression in isolated adult rat cardiomyocytes by immunofluorescence (Fig. 8B). There was a significant increase in Cx43 levels in cardiomyocytes treated with conductive polymer- GO-Au. In this regard, previous studies have demonstrated that decreased Cx43 expression after an MI was associated with deterioration of electrical conduction and ventricular function^49^. In diseased myocardial tissue, reduced Cx43 levels are associated with slow conduction and cardiac arrhythmias. In our study, there was a significant increase in Cx43 levels in the scaffold implanted animals after a myocardial infarction. Therefore, improved conduction velocity and cardiac contraction in our studies after scaffold implantation might have been due to the upregulation of Cx43 expression.

In conclusion, current study reports synthesis and application of a chitosan-graphene oxide-gold nanoparticles based biodegradable conductive scaffold for cardiac repair. The scaffold is biocompatible, electrically conductive, and it displayed controlled degradation properties. Even though in our *in vivo* experiments (see Supplementary Fig. 2) we found that at the end of 5 weeks the patch was present in the heart. However, our *in vitro* degradation studies suggest that the scaffold will dissolve slowly after implantation. However, in future to construct clinically relevant conductive polymer patches, more elaborative studies are needed to monitor the long-term fate of implanted scaffold *in vivo* and it’s long term effects on cardiac function.

## Methods

### Graphene oxide preparation

Graphene oxide (GO) nanosheets were synthesized from graphite powder as described in detail in supplementary methods.

### Preparation of graphene oxide-gold-nanoparticle (GO-Au) composite

To synthesize GO-Au composites, 20 mg GO powder was dispersed in 50 ml of ultra-pure water under sonication for 1 hr at room temperature. Gold precursor solution containing 20 mg chloroauric acid (HAuCl_4_) was added in drops and stirred at room temperature. Then sodium citrate (5 ml) was added in drops to the mixture and sonicated for 1 hr. The solution was then heated to 80 °C for 1 hr under continuous stirring conditions until a dark red wine colored solution was formed. Finally, the contents were centrifuged at 6000 rpm to remove free gold nanoparticles from the solution and washed thrice with distilled water and dried in an oven at 60 °C for 12 hr.

### Fabrication of chitosan-GO-Au scaffold

The chitosan (CS) and GO-Au composite scaffold was fabricated by freeze drying method to obtain a porous morphology. Briefly, chitosan (SigmaAldrich, CA) 2% (w/v) was dissolved in 1% (v/v) acetic acid (SigmaAldrich, CA) under stirring conditions overnight and centrifuged to remove the undissolved chitosan flakes. GO-Au nanocomposite was added to the chitosan solution at 0.1%, 0.25% and 0.5% (w/v) and stirred for 2 hr. The mixture was sonicated for 30 min and stirred continuously overnight to obtain a homogenous suspension. CS/GO-Au mixture was casted in 24 well plates and frozen at −80 °C overnight. The nanocomposites were then neutralized in 1% NaOH (w/v) and washed several times with distilled water to adjust the pH to 7.4. The nanocomposites were then lyophilized for 16 hr to obtain the final scaffolds.

### Physico-chemical characterization of the scaffold

The physico-chemical characterization (morphology and microstructure) of the lyophilized scaffolds was performed by SEM, TEM, X-ray diffraction (XRD) and FT-IR analyses as described in detail in supplementary methods.

### Swelling ability and biodegradation

The swelling ability and the rate of biodegradation of the scaffold were measured using standard procedures as described in supplementary methods.

### Electrical conductivity measurement

The electrical conductivity of the CS, CS-0.5% GO and CS-0.5% GO-Au (chitosan containing 0.5% GO-Au) scaffolds was measured by four-probe method with 2 contact pairs. The conductivity was then expressed as S/cm and calculated by the following equation. D represents the distance between the measurement probes in mm, V is the corresponding voltage in mV and I represent the current supplied in mA.

$${\rm{Conductivity}}=\frac{1}{[2\pi D(\frac{V}{I})]}$$

### *In vitro* biocompatibility of the scaffold

The biocompatibility of the scaffold was assessed using rat smooth muscle cells, mouse fibroblasts and human iPS derived cardiomyocytes as described in detail in supplementary methods.

### *In vivo* studies

#### Animals

Adult male Wistar rats (140–150 g), (n = 30) were used for this study. All animals were purchased and kept in the animal care facility of the National Institute of Ophthalmological Research, Cairo University. The experimental protocol and procedures were approved by the Institutional Animal Care and Use Committee of the Cairo University. All the methods were carried out in accordance with the guidelines from Cairo University.

#### Myocardial infarction model and scaffold implantation

Animals were placed in right decubitus position over a heating blanket. The chest was shaved and disinfected with betadine. Left lateral thoracotomy was performed and hearts were visualized using self-retaining retractor, the pericardium was gently removed. Myocardial infarction (MI) was induced by ligating the left anterior descending coronary artery. Immediately after ligation, scaffold (sterile scaffold which was cut into 5 × 2 mm round pieces) was sutured to the site of infarction using 7.0 polypropylene suture. The chest was closed using 4.0 nylon interrupted sutures.

Cardiac function was evaluated by echocardiography (ECHO) at baseline, after 1 week and 5 weeks of scaffold implantation. Two–dimensional and M-mode recording of short axis view was performed using ultra-sonographic machine (Samsung Madison, SONOACE-R3). The following measurements were recorded: left ventricular internal dimension at diastole (LVIDD), left ventricular internal dimension at systole (LVIDS), % fractional shortening (FS%) and % ejection fraction (%EF).

#### Electrocardiogram measurements

After 5 weeks of scaffold implantation in the infarcted heart, electrocardiograms (ECG) were recorded by non-invasive method. Animals were supported in cotton jacket and bandage in supine position, local anesthesia was applied on the four extremities. After that animals were allowed to equilibrate for 5 minutes. Five minute recordings were obtained using ECG switch box MLA0114 amplified by Boi Amp ML408 and analyzed using Lab Chart 7 software (AdInstruments) and the 5 min sequences were averaged to obtain standard ECG wave intervals and durations.

#### CD4^+^ and CD8^+^ T cell infiltration in the heart

To assess host immune response against implanted scaffold, we performed immunohistochemistry and evaluated CD4^+^ and CD8^+^ T cell infiltration in the myocardium at 5 weeks after scaffold implantation. Briefly, heart tissue samples were fixed in formalin and cut into 5-µm thick sections on poly-lyisne coated slides. After removing the wax, sections were rehydrated using different concentrations of ethanol (100%, 95%, 70%, 50%) and washed with 1X PBS for 10 min. After blocking with 1% horse serum for 30 min, sections were incubated with anti-CD4 or anti-CD8 antibody (Cedarlane, CL003AP, CL004AP) at 1:100 dilution overnight at 4 °C. The secondary antibody conjugated with alexa fluor 647 (Life-Technologies, CA) was applied to the sections (dilution; 1:150) for 30 min at room temperature. The slides were then mounted with anti-fade mounting media containing DAPI (Abcam, CA). Next day, the images were recorded using Cytation 5 imaging reader and quantified using Image J software.

#### *Ex-vivo* Langendorff heart perfusion

At the end of *in vivo* experiments, cardiac contractility was measured in isolated hearts using Langendorff apparatus, as described in supplementary methods.

#### Connexin 43 expression

Connexin 43 levels were measured in cardiac tissue samples (by immunohistochemistry) and in isolated cardiomyocytes (by immunocytochemistry) as described below.

#### Immunohistochemistry

Cardiac tissue samples from different groups were fixed in formalin and processed as described above (for CD4^+^ and CD8^+^ T cell staining). The sections were incubated with anti-connexin 43 (Cx43) antibody (Abcam, CA) at 1:100 dilution overnight at 4 °C. The secondary antibody conjugated with alexa fluor 647 (Life-Technologies, CA) was applied to the sections (dilution 1:500) for 30 min at room temperature. The sections were washed with 1X PBS and incubated for 10 min with F-actin antibody conjugated with alexa fluor 488 (ThermoFisher Scientific, CA) and washed with 1X PBS. The slides were then mounted with anti-fade mounting media containing DAPI (Abcam, CA). Next day, the images were recorded using Cytation 5 imaging reader and quantified using Image J software.

#### Immunocytochemistry

Connexin43 expression was also measured in adult rat cardiomyocytes. Cells were isolated from normal adult Sprague Dawley rats (250–300 g) using Langendorff apparatus and plated in laminin coated (20 µg/ml) culture plates. Unattached and dead cells were removed after 2 hr and attached viable cardiomyocytes were incubated overnight in M199 medium supplemented with 10% FBS at 37 °C under 5% CO2. Cardiomyocytes were treated with 0.5% GO-Au composite for a period of 24 hr. Cells cultured without GO-Au composite served as control. After the treatment period, cardiomyocytes were washed with PBS and fixed in 4% paraformaldehyde for 20 min. Fixed cells were probed (overnight) with anti-connexin-43 antibody (1:500) at 4 °C. Next, the cells were incubated with alexa fluor 647 conjugated goat anti-rabbit (1:1000) secondary antibody. Cells were then stained with Phalloidin (Thermo Fisher Scientific, CA) at a dilution of 1:1000 followed by counter staining with DAPI (Sigma Aldrich, CA). Finally, the images were recorded using Cytation 5 imaging system.

#### Statistical analysis

Data were analyzed with GraphPad Prism software and expressed as mean ± SD. The comparisons between two groups were made with a two-tailed Student’s *t*-test. The comparisons among multiple groups were performed with either one-way or two way ANOVA. When *F* values were significant, group differences were specified with Tukey or Bonferroni post-hoc test. The differences were considered statistically significant when *p* < 0.05.

## Electronic supplementary material

Supplementary File

## Data Availability

All data generated or analysed during this study are included in this published article (and its Supplementary Information files).
