# Supplementary material for: Graphene Oxide-Gold Nanosheets Containing Chitosan Scaffold Improves Ventricular Contractility and Function After Implantation into Infarcted Heart
Source: Sci Rep. 2018 Oct 10;8:15069. doi: 10.1038/s41598-018-33144-0 (PMC6180127; doi:10.1038/s41598-018-33144-0)
Supplement: Supplementary file 1 — Supplementary File [file 41598_2018_33144_MOESM1_ESM.pdf]

Supplementary File

Graphene Oxide-Gold Nanosheets Containing Chitosan Scaffold Improves Ventricular Contractility and Function After Implantation into Infarcted Heart

*Sekaran Saravanan[1], Niketa Sareen[1], Ejlal Abu-El Rub[1], Hend Ashour[2], Glen Lester Sequiera[1], Hania I. Ammar[2], Venkatraman Gopinath[3], Ashraf Ali Shamaa[2], Safinaz Salah Eldin Sayed[2], Meenal Moudgil[1], Jamuna Vadivelu[3], Sanjiv Dhingra[1]**

1 Regenerative Medicine Program, Institute of Cardiovascular Sciences, St. Boniface Hospital Research Centre, Department of Physiology and Pathophysiology, University of Manitoba, Winnipeg, Canada

2 Department of Physiology, Biochemistry and Histology, Faculty of Medicine, Cairo University, Egypt

3 Department of Medical Microbiology, Faculty of Medicine, University of Malaya, Kuala Lumpur, Malaysia

***Correspondence:**
Sanjiv Dhingra, PhD
Regenerative Medicine Program
Institute of Cardiovascular Sciences,
St. Boniface Hospital Research Centre
University of Manitoba
R-3028-2, 351 Tache Avenue, Winnipeg, R2H2A6, Canada
Email: sdhingra@sbrc.ca

Supplementary methods

Graphene oxide preparation: Graphene oxide (GO) nanosheets were synthesized from graphite powder using an improved method reported by Marcano et al.[1]. Briefly, 1 g of graphite powder (SigmaAldrich, CA) was added to H_2SO_4/H_3PO_4 mixture (9:1 v/v) and stirred to form a black viscous solution to which 6 g of $KMnO_4$ was added and stirred at 50°C overnight. At the end of reaction period, the mixture was allowed to cool down to room temperature and poured onto 200 ml of ice and mixed gently. 30% H_2O_2 was added in drops with continuous stirring. The mixture was centrifuged and washed multiple times with water, HCl and absolute ethanol to obtain purified graphene oxide dispersion. The final product was collected through centrifugation and lyophilized to obtain graphene oxide powder. The physico-chemical characterization of the material was done by transmission electron microscopy (TEM), fourier transform infrared spectroscopy (FT-IR), and Raman Spectroscopy as described previously[2,3].

Physico-chemical characterization of the scaffold: The lyophilized scaffolds were cut with surgical blade to expose internal porous microstructure and sputter coated with gold for SEM analysis at 10 kV using FEI Quanta E-SEM. For X-ray diffraction (XRD) and FT-IR analyses, the scaffold discs were grounded into powder in liquid nitrogen. The X-ray diffractogram were recorded between 0°-80° at "2θ angle" at a scan speed of 1° min^{-1} using PANalytical X'Pert Pro MPD High Temperature Powder X-ray Diffractometer with an operating voltage of 40 kV. Functional group interaction between individual components of the scaffolds was assessed by FTIR spectrum (Perkin Elmer) with a scan range from 4000 to 450 cm^{-1}.

Measurement of swelling abilities of the scaffold: Swelling studies were performed with freeze dried scaffolds, we used 100mg of scaffold in different groups (recorded as Mi). The scaffolds were hydrated by immersing in 10ml of 1X PBS and 15% FBS supplemented DMEM (Life-Technologies, CA) for different time points (1 h, 24 h and 48 h) at 37°C; 5% CO2 in incubator. After hydration

process at different time points, the scaffolds were surface blotted to remove adsorbed ions on the surfaces and the wet weights were recorded as Mf. The swelling percentages of the scaffolds were then calculated using following equation:

$$\text{Swelling percentage (SW\%)} = (\text{Mi-Mf})/(\text{Mi}) \times 100$$

Measurement of biodegradation rate of the scaffold: To measure degradation rate of scaffolds, 100mg (W0) of scaffold was incubated in 10ml of DMEM containing lysozyme (5mg/10ml of DMEM) (Sigma Aldrich, CA) for 5 weeks at 37°C. At the end of incubation period, the scaffolds were removed and freeze dried to record the final weight (Wf). The percentage of degradation of the scaffolds was then calculated by the equation:

$$\%\text{BD} = (\text{W0-Wf})/\text{Wf} \times 100$$

Electrical conductivity measurement:

In vitro biocompatibility of the scaffold: To assess biocompatibility of the scaffold with heart cells, we used rat smooth muscle cells, mouse fibroblasts and human iPS derived cardiomyocytes as described in the following section;

Compatibility with rat smooth muscle cells: The experimental protocol and procedures were approved by the institutional animal care and use committee of the University of Manitoba. Smooth muscle cells (SMCs) were isolated from rat aortas (Sprague Dawley rats) using 0.2% trypsin (Gibco Biosciences, Dublin) and 0.1% collagenase (Worthington Biochemical Corp. New Jersey) digestion as described in our previously published study[4]. The cells were cultured in Dulbecco's Modified Eagle's Medium (Gibco Biosciences, Dublin) supplemented with 15% FBS, 1% penicillin/streptomycin at 37°C in a humidified incubator with 5% CO_2. The medium was replenished every 2 days and cells were grown to confluency. Scaffold solution including naïve chitosan (control group) and chitosan-0.5%GO-Au composite were poured into 24 well culture dishes

in the form of a thin membrane and dried overnight at 37°C with the plates uncovered and devoid of humidity and carbon dioxide. The scaffolds were then neutralized with 0.1M NaOH solution and washed 3 times with PBS and air dried under sterile conditions. Followed by ethanol sterilization, UV treatment for 15 min, and then medium equilibration for 2 hr. Equal number of SMCs (in 400µl of DMEM containing 15% FBS) were added to each well containing scaffold. Uncoated wells served as control. After 48 hr of incubation the medium was extracted and MTT assay was carried out to measure the growth of the cells (an indirect measure of biocompatibility) as described in our previous study[5]. Cells treated with 0.1% triton served as positive control. The experiments were carried out in triplicates.

Biocompatibility was also assessed by fluorescein diacetate (FDA) staining (SigmaAldrich, CA). FDA is a very good indicator of cellular morphology, the staining was performed as described previously[5]. Briefly, rat SMCs were grown in the plates coated with chitosan or chitosan-GO-Au for 48hr. Uncoated wells served as control. After initial incubation, cells were treated with FDA solution (35 µg/ml) for 24 hr. At the end of the treatment period images were captured with Cytation5 imaging system (Bio-Tek Inc.) to analyze the cellular morphology.

Compatibility with mouse fibroblasts: PMEF-CFL-P1 mouse embryonic fibroblasts (Millipore Sigma, Canada) were cultured in DMEM (Gibco Biosciences, Dublin) supplemented with 10% fetal bovine serum (Gibco Biosciences, Dublin), 100 units/ml penicillin G and 0.1 mg/ml streptomycin (Life Technologies, California) at 37°C with a 5% CO_2 in air atmosphere. To assess compatibility the cells were grown in the culture plates coated chitosan or chitosan-GO-Au for 48hr as described in the previous section. Biocompatibility was assessed by MTT assay and FDA staining as described above.

Compatibility with human iPSC derived cardiomyocytes: Peripheral blood mononuclear cells isolated from human blood (collected from healthy individuals) were reprogrammed to iPSC using

CytoTune™-iPS 2.0 Sendai Reprogramming Kit (ThermoFisher Scientific, US). All the protocols and procedures were approved by the University of Manitoba's Research Ethics Board (REB). Once the iPSC clones were established, they were differentiated into cardiomyocytes[6]. The iPSC derived cardiomyocytes (iPSC-CMs) were dissociated with TryPLE™ Express (Life Technologies, US) and plated at a density of 3000cells/well of a 96 well plate on different surfaces – normal tissue culture surface, Matrigel and CS-0.5%GO-Au scaffold. Cells were allowed to attach for 48hr. After that the cells were manually counted under phase-contrast microscope. The percentage of attachment was counted as number of cells attached after 48hr divided by number of cells seeded multiplied by 100.

Ex-vivo Langendorff heart perfusion: At the end of *in vivo* experiments, the animals were anaesthetized and hearts were rapidly excised and immediately placed in ice cold Kreb-Henseleit (KH) heparinized solution. The ascending aorta was then cannulated and placed along the perfusion line of a non-recirculating constant flow Langendorff apparatus (Radnotti, Harvard apparatus, USA) at 37ºC. The duration between excision and perfusion of the hearts didn't exceed one minute. Hearts were then perfused using KH buffer (Sigma Aldrich, MO, USA). Perfusion was maintained at a constant flow of 16ml/min at 37ºC and aerated with gas mixture (95%O2, 5%CO2). Hearts were allowed to beat spontaneously throughout the experiment. To determine left ventricular pressure, a latex balloon was inserted into the left ventricle through an incision in the left atrial appendage. The balloon was tied securely into place and filled with saline to give an end-diastolic pressure of ~ 10-15mmHg. The intraventricular balloon catheter was connected to a pressure transducer. Left ventricular pressure and heart rate were monitored continuously and recorded. Digital analysis of the wave was performed and displayed by an electronic polygraph (NEC-San-ei, 2238, Tokyo, Japan). The maximum and minimum rate of pressure rise dp/dt max and dp/dt min (two sensitive indices for contractility) were calculated.

References

1. Marcano, D. C. *et al.* Improved Synthesis of Graphene Oxide. *ACS Nano* **4,** 4806–4814 (2010).
2. Viinikanoja, A., Kauppila, J., Damlin, P., Suominen, M. & Kvarnström, C. In situ FTIR and Raman spectroelectrochemical characterization of graphene oxide upon electrochemical reduction in organic solvents. *Phys. Chem. Chem. Phys.* **17,** 12115–12123 (2015).
3. Wilson, N. R. *et al.* Graphene Oxide: Structural Analysis and Application as a Highly Transparent Support for Electron Microscopy. *ACS Nano* **3,** 2547–2556 (2009).
4. Dhingra, S. *et al.* Modulation of Alloimmune Responses by Interleukin-10 Prevents Rejection of Implanted Allogeneic Smooth Muscle Cells and Restores Postinfarction Ventricular Function. *Cell Transplant.* **24,** 1013–1029 (2015).
5. Saravanan, S., Vimalraj, S., Vairamani, M. & Selvamurugan, N. Role of Mesoporous Wollastonite (Calcium Silicate) in Mesenchymal Stem Cell Proliferation and Osteoblast Differentiation: A Cellular and Molecular Study. *J. Biomed. Nanotechnol.* **11,** 1124–1138 (2015).
6. Burridge, P. W. *et al.* Chemically defined generation of human cardiomyocytes. *Nat. Methods* **11,** 855–860 (2014).

Supplementary Figure 1. Connexin 43 expression in myocardial tissue sections: (A) Photomicrographs of rat myocardial sections (immunohistochemistry) in control group (MI) and scaffold (CS-GO-Au) treated group. **(B)** Quantification of connexin 43 (Cx 43) expression in infarct area and normal myocardial tissue. The ratio of Cx 43 expression in infarct area/normal tissue increased in scaffold treated animals compared to control group. (magnification 4X, stitched images). (F-actin- green; Connexin 43- red; DAPI- blue). *$P<0.05$ compared to control group. (n=5) Data are expressed as mean±SD.

Supplementary Figure 2. Pictures showing scaffold (blue arrows) at 5 weeks after implantation in the infarcted hearts in two different animals from scaffold treated group.

Supplementary Figure 1

(A)

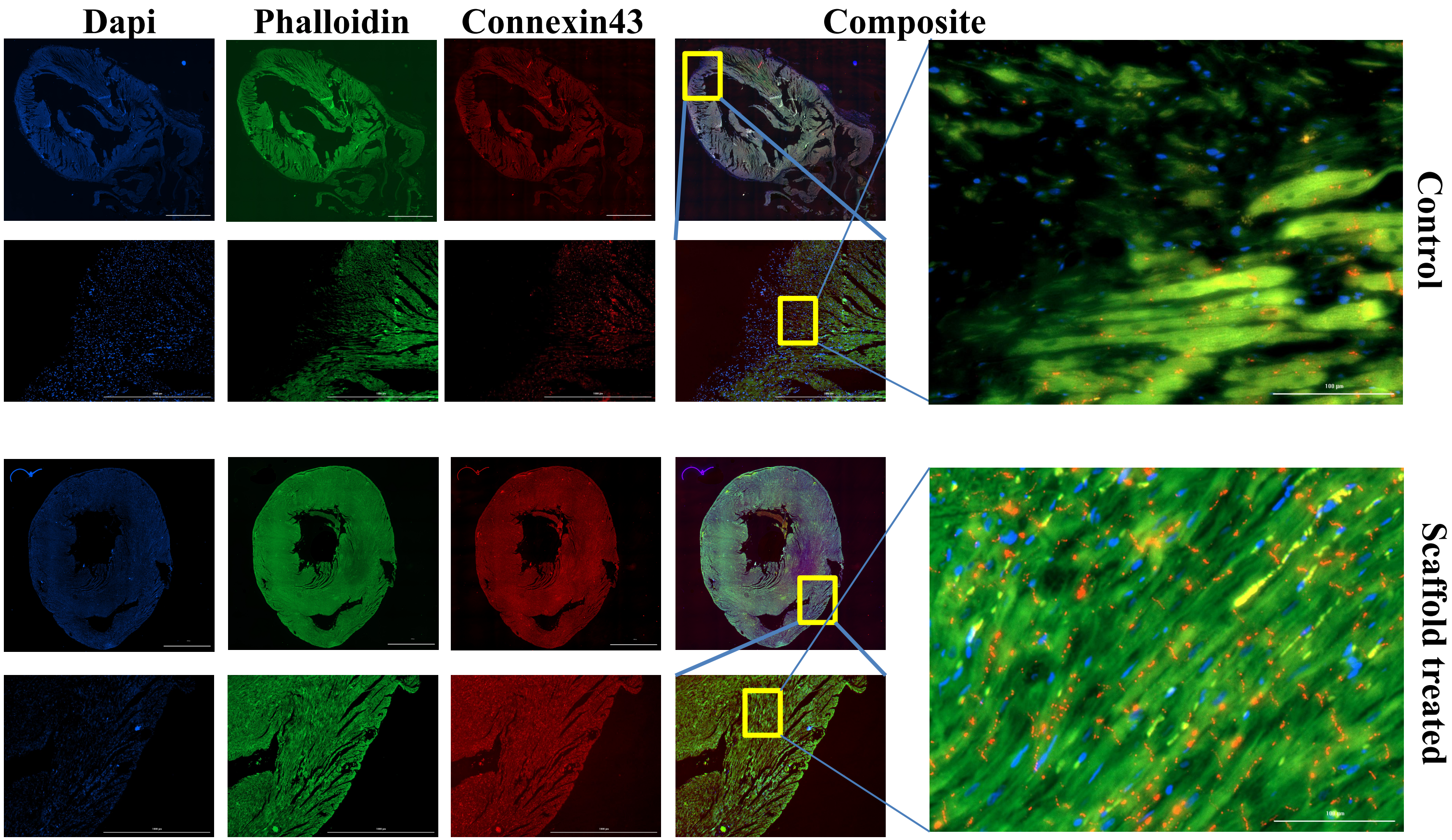

(B)

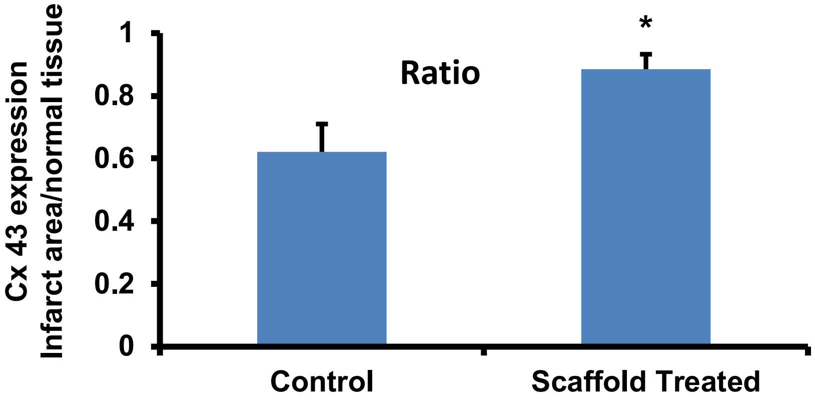

Supplementary Figure 2

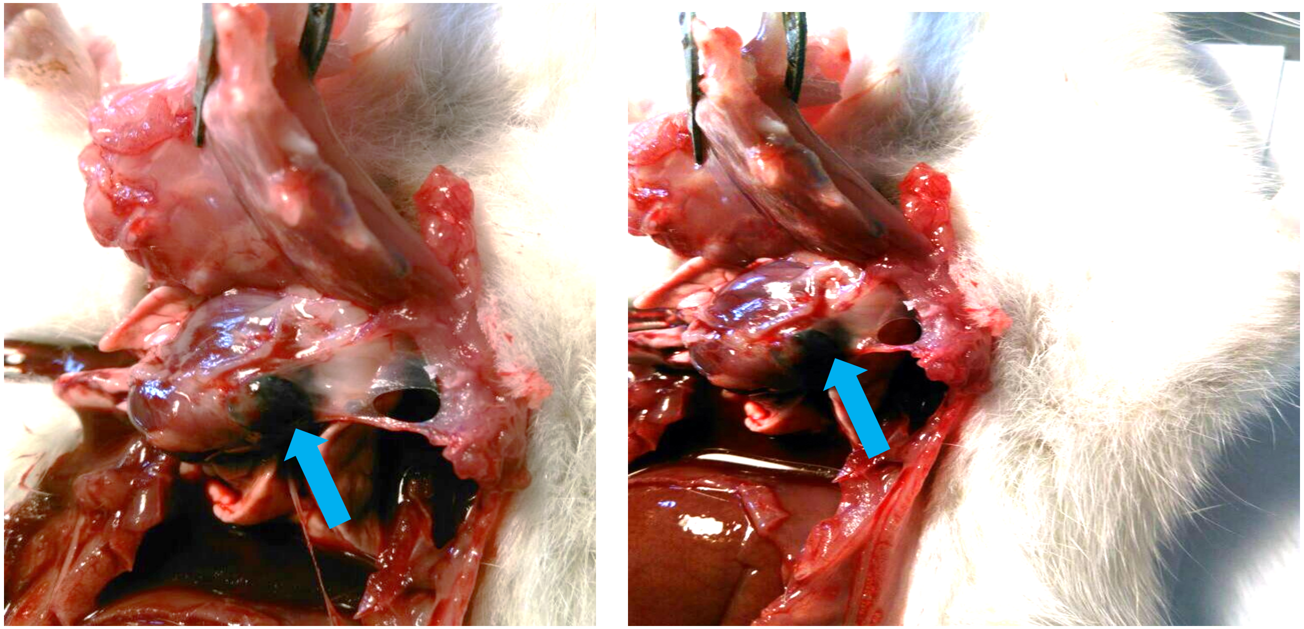

Hearts from two different animals showing scaffold (blue arrows) at 5 weeks after implantation in scaffold treated group
